# Parapharyngeal abscess: A difficult diagnosis in younger children

**DOI:** 10.1002/ccr3.2209

**Published:** 2019-05-10

**Authors:** Sathyanarayan Sudhanthar, Anjali Garg, Jonathan Gold, Olga Napolova

**Affiliations:** ^1^ Department of Pediatrics and Human Development, College of Human Medicine Michigan State University East Lansing Michigan; ^2^ Present address: UH Rainbow Babies and Children's Hospital 11100 Euclid Avenue, RB&C Suite 838 Cleveland MI 44106‐1716

**Keywords:** ear, nose, and throat, infectious disease, patient education, pediatrics and adolescent medicine

## Abstract

Parapharyngeal abscess is a difficult diagnosis to make in the pediatric population. Children <3 years of age present with fever, often the only sign at the initial presentation, thus causing a delay in diagnosis until the patient presents with more focal findings, such as a painful neck mass. Repeated follow‐up visits with the patient until the diagnosis is confirmed are critically important to prevent complications.

## 1 BACKGROUND

The parapharyngeal region is the most common site of deep neck infections.^1^, ^2^ In addition to fever, the presenting symptoms in a patient with a parapharyngeal abscess include limited neck movement and neck pain.^1^, ^3^, ^4^, ^5^ These symptoms are usually difficult to observe in a child <3 years of age, as they are not able to verbalize the location and severity of their pain. Other common presenting symptoms, such as a sore throat, swelling, and dysphagia, present inconsistently and are therefore unreliable. Hence, in most cases, fever may be the only sign at the initial presentation, thus causing a delay in diagnosis until the patient presents with more focal findings, such as a neck mass or pain.^1^ A delay in diagnosis may lead to an increase in complications and may eventually warrant surgical treatment.^5^

## 2 CASE PRESENTATION

Our patient is a 2‐year‐old male who presented with a 1‐day fever (Temperature: 103.6°F), cough, congestion, and inadequate oral intake. The patient's history was obtained from the patient's mother. She denied any signs of vomiting, abdominal pain, diarrhea, respiratory distress, ear pain, or a sore throat. Additionally, the patient showed signs of dehydration. Upon an initial examination, he was agitated but consolable. An examination of his ears revealed no external deformities; his canals were patent and without inflammation, and his tympanic membranes were intact, gray, translucent, and mobile. His nose showed no external deformities, and the nares were patent. His nasal turbinates were erythematous, but no inflammation was exhibited. His oral structures were normal for a child of his age, and the mucous membranes were moist and pink, without any lesions or exudates. His teeth did not have any dental caries. His neck was supple, and no cervical lymphadenopathy was present. The rest of his physical examination also revealed negative findings. The patient's fever and irritability warranted initial laboratory tests, including a rapid influenza test, a complete blood cell count (CBC), a C‐reactive protein (CRP) test, and a blood culture. Abnormal values included leukocytosis that exhibited a left shift (26 500 cells/µL) and an elevated CRP (24.7 mg/L).

Due to his elevated CBC and CRP, the patient was called back into the office for a re‐evaluation the next day. His physical examination revealed new findings of erythematous tonsils with a midline uvula, as well as left and right posterior cervical nodes. The tenderness of the nodes was difficult to decipher because the patient was irritable throughout the examination. The hydration status of the patient had improved from the previous night. The rest of his physical examination revealed similar findings as the previous day. A urinalysis was obtained via catheterization and revealed negative results. Since his elevated WBC count raised concerns of the possibility of a bacterial infection, an intramuscular ceftriaxone injection was given. The patient was sent home with instructions to the parents to report any changes in his symptoms or any reactions to the antibiotics. Subsequent tests for CBC and CRP, which were obtained on the 4th day of the patient's symptoms, were still elevated, although the levels had improved (20 000 cells/µL and 14.6 mg/L, respectively). The patient initially responded well to the antibiotics; however, his fever returned, with a temperature of 103.8°F. He was seen again in the office on the 6th day of his symptoms. At this point, he presented with a new finding of left neck swelling. He had cervical asymmetry, and his left tonsil was deviated toward the midline, due to soft tissue swelling.

## 3 DIFFERENTIAL DIAGNOSIS

Initially, our patient only presented with a fever and a lack of focal findings, which resulted in our primary diagnosis being a viral infection vs a bacterial infection, with the latter diagnosis being included due to leukocytosis. The negative rapid influenza test ruled out the most likely etiology of a viral infection. The most worrisome differential diagnoses of bacterial etiology in his age group are bacteremia, a urinary tract infection, and meningitis. The blood culture that was obtained on the first day of the presentation of symptoms was negative. With this result, and in addition to the negative urinalysis results, we were able to rule out the diagnoses of bacteremia and a urinary tract infection, respectively. Meningitis was not considered because the patient had no meningeal signs, and he was consolable, although irritable. Initially, the lack of substantial cervical lymphadenopathy and a Centor score of 1 for fever negated the need for a Strep rapid antigen detection test, which reduced our concern for the presence of pharyngitis. Therefore, testing was performed to identify potential viral causes, such as Influenza, which was ruled out via a rapid influenza test. We maintained a high degree of clinical suspicion of an underlying bacterial etiology, given that his age posed a diagnostic barrier. As his illness progressed, he began to show signs of lymphadenopathy, followed by unilateral neck swelling.

## 4 TREATMENT

**Figure 1:**
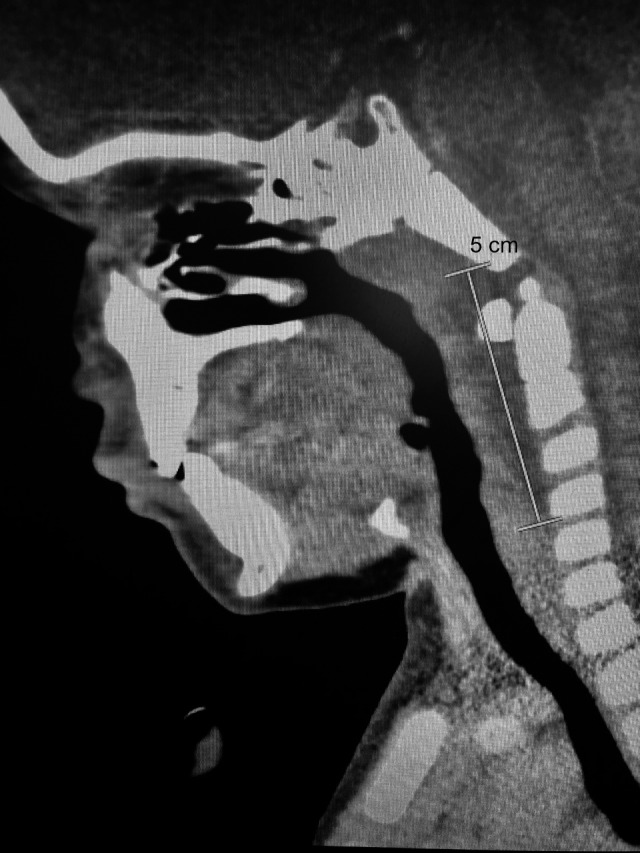
The craniocaudal extent of the retropharyngeal fluid is 5 cm from the clivus through to the bottom of C4

**Figure 2:**
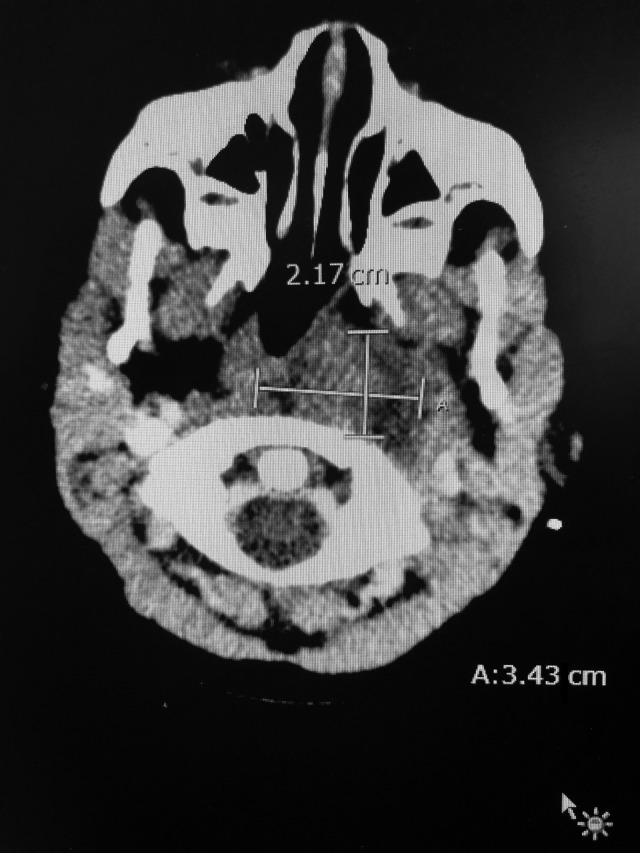
Section of the CT shows the fluid present in the nasopharyngeal region is 3.4 cm transverse by 2.2 cm AP

**Figure 3:**
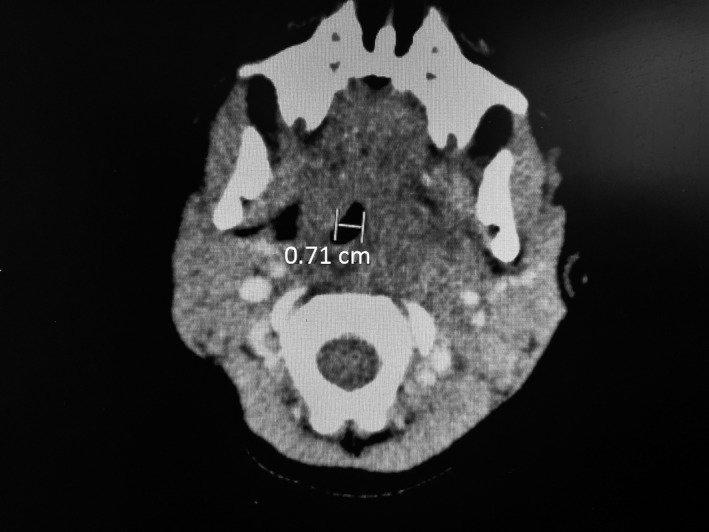
The mass effect upon the airway which is displaced from the left to right. The airway is not completely effaced, though it is significantly impacted. It is narrowed to approximately 7 mm at its point of maximum narrowing

He was admitted to the hospital for further management after the presentation of unilateral nuchal swelling. A CT scan of the neck was ordered and showed a left‐sided nasopharyngeal level abscess that extended into the left parapharyngeal space posteriorly within the tonsillar soft tissue (Figures 1 and 2). An area of hypodensity indicated that there was retropharyngeal fluid collection, which correlates with a deviation of the airway toward the right (Figure 3). A Streptococcus Group A throat swab and subsequent PCR of the swab was ordered and was found to be positive. He was started on IV ampicillin‐sulbactam.

## 5 OUTCOME AND FOLLOW‐UP

The patient showed improvement with medical management; therefore, a surgical intervention was deemed to be unnecessary. He was discharged after 3 days and completed a 7‐day course of oral amoxicillin‐clavulanic acid. The patient was able to fully complete treatment and experienced no further complications. He was seen in the office 2 weeks later and had made a full recovery.

## 6 DISCUSSION

Parapharyngeal abscesses are incorporated into a group of infections known as deep neck infections.^1^, ^2^ These infections generally occur due to nontraumatic reasons in young children, such as a prior throat infection or an infection of dental origin that has seeded into the deeper tissue structures and lymph nodes.^4^, ^6^ Cultures of abscesses demonstrate that mixed (anaerobic and aerobic) pathogens are the most common causes of infection, followed by Group A Streptococci.^1^, ^4^, ^7^ Several cases have also reported *Staphylococcus aureus* to be a common cause.^4^, ^7^, ^8^ However, while abscess cultures are usually positive, the majority of blood cultures are negative, thus adding to the challenge of these diagnoses.^1^, ^4^ The primary physical examination finding that should indicate to the physician a diagnosis of a parapharyngeal abscess is limited neck movement.^3^, ^4^ These patients will often hold their neck in a neutral position and will only use their eyes to look toward objects that are out of their field of view.^4^ This finding was difficult to ascertain in our patient due to his young age. His irritability upon the examination masked any potential limitations in neck movement, and without any other focal findings, a proper diagnosis was difficult to make. Additionally, he was unable to verbalize where his pain was located. Therefore, the maintenance of a high degree of clinical suspicion was significant, and the repeated evaluation of our patient allowed us to eventually diagnosis the problem. A CT scan of the neck, as well as presenting symptoms of fever, unilateral cervical lymphadenopathy, and limited neck movement, aids in the diagnosis of this condition.^3^, ^4^ Treatment options include intravenous (IV) antibiotics with or without surgical drainage. Antibiotic treatment for hospitalized patients includes the use of Clindamycin or Ampicillin‐Sulbactam.^4^, ^8^ If there is no improvement with IV antibiotic treatment within 48 hours, then the surgical drainage of the abscess is indicated.^5^, ^6^, ^7^ With the advent of the CT scan, diagnoses of parapharyngeal abscesses are being made at earlier times, thus increasing the rate of successful medical treatments without the need for surgical interventions.^4^, ^6^ A high degree of suspicion is still needed to perform a CT scan, given the radiation exposure risk for young children. Additionally, parapharyngeal abscesses are dangerous, due to their location and ability to obstruct the airway, which makes a timely diagnosis important.^4^ An internal jugular vein thrombosis, cranial nerve (specifically IX and XII) dysfunction, or cervical chain dysfunction are reported complications.^2^, ^5^ The fact that we followed our patient closely in the clinic allowed us to track his disease progression, which then allowed us to make a diagnosis before any complications occurred. Through multiple interactions, we were able to observe the development of his focal symptoms. Therefore, a diligent diagnosis is essential in young children who present with vague symptoms and no discernable focuses on infection.

## 7 LEARNING POINTS

- Pediatric patients who present with a parapharyngeal abscess are difficult to clinically diagnose. Especially difficult are children <3 years old who are not able to appropriately verbalize their symptoms.
- A CT scan and clinical symptoms are diagnostic parameters for diagnoses of parapharyngeal abscesses, but a patient may often only present with a fever as his or her initial symptom.
- Repeated interactions with the patient, until a diagnosis is made, are essential to prevent complications.

## CONFLICT OF INTEREST

None declared.

## AUTHOR CONTRIBUTIONS

All the authors were involved in the patient care when the patient was scheduled for regular follow‐ups. AG: wrote the first draft of the manuscript and SS, JG, and ON: were involved in the revision of the manuscript. SS: involved in getting the consent from the family and their perspective. SS and AG: prepared a poster presentation of this case report for the AAP NCE conference. AG and SS: acquired the images for this case report and changed them to high resolution.

## PATIENT/PARENT PERSPECTIVE

“At first, we thought it was just a fever and likely some virus going around. My son was miserable and will not eat or drink properly. We were giving him fever reducers, but still, the fever will start coming up. After he was seen the first time, we were keeping a close eye and were getting worried that he was not getting better. Once we started noticing some swelling in his neck, we were just very concerned. Even after he got the antibiotic shot, he was still not getting better, and that's when we rather thought there is something more going on. We are thankful that all the three times he was seen he was examined and his primary care doctors made proper decisions. After he was admitted to the hospital and had antibiotics and fluids; he was beginning to perk up. When the doctors in the hospital showed us where the pus was hiding, we could understand why his fevers kept coming. We are just glad that he is doing fine now.”
